# Shared decision-making in depression: From a generalized ethical imperative to a personalized approach? A narrative review

**DOI:** 10.1192/j.eurpsy.2026.12221

**Published:** 2026-05-29

**Authors:** Koen Demyttenaere, Elke Heirman, Stefano Barlati, Anthony J. Cleare, Alessandra Minelli, Inga Stonner, Becci Strawbridge, Bernhard T. Baune

**Affiliations:** 1 Neurosciences, Katholieke Universiteit Leuven, Leuven, Belgium; 2 Universitair Psychiatrisch Centrum KU Leuven – Campus Lubbeek: Katholieke Universiteit Leuven, Leuven, Belgium; 3 University of Brescia: Universita degli Studi di Brescia, Brescia, Italy; 4 Department of Psychological Medicine, King’s College London, London, UK; 5 Department of Molecular and Translational Medicine, University of Brescia, Brescia, Italy; 6 Genetics Unit, IRCCS Istituto Centro San Giovanni di Dio Fatebenefratelli, Brescia, Italy; 7 University of Munster: Westfalische Wilhelms-Universitat Munster, Germany; 8 Institute of Psychiatry at the Maudsley: King’s College London Institute of Psychiatry, UK

**Keywords:** depression, personalized medicine, shared decision-making, theapeutic alliance, evidence-based medicine

## Abstract

**Background:**

While both evidence-based medicine as well as personalized patient-centered medicine are glorified, clinical practice seems to be only partially influenced by guidelines or by patients’ views. Against this background, shared decision-making (SDM) became a hype but remains an ill-defined concept: it can be considered as an umbrella concept covering therapeutic alliance, physician and patient preferences, information giving, information retrieving, information receiving, patient involvement in goal setting, decision-making, and treatment choice.

**Methods:**

A narrative review based on a literature search in PubMed and PsycINFO.

**Results:**

SDM in depression should move away from an ethical imperative where “one size fits all” toward a personalized approach, tailoring the medical encounter to the physician’s and patient’s expectations (knowledge, need for knowledge, and desire for degree of SDM) and to the patient’s clinical status. SDM cannot be conceived as a “snapshot” since it is a dynamic process where adjustments should be made depending on the growth of the doctor–patient alliance, the changes in expectations, and the changes in clinical status. The clinician should be more aware of barriers to effective implementation of SDM in him or herself, in the patient. The different definitions of SDM and the many reported SDM interventions (having different ingredients) hamper final conclusions on the effect of SDM on outcomes.

**Conclusions:**

This narrative review aims to critically examine SDM in depression and proposes to conceptualize it as a dynamic, relational, and personalized process rather than a universal ethical standard.

## Introduction and background

### A broader perspective on evidence-based practice

The last decades saw evidence-based medicine and clinical guidelines becoming the predominant and idealized model in all fields of medicine, including psychiatry, both in graduate and postgraduate teaching programs. Recently, and for multiple reasons, hesitations have been expressed concerning this trend [1]. First, a depressed patient population is heterogeneous, and the “mythical” average patient does not exist. Second, relying on randomized clinical trials (RCTs) results as the gold standard is debatable since inclusion and exclusion criteria make that only 10–20% of clinical practice patients can be enrolled in RCTs [2, 3]. Third, different guidelines show differences since they are only partially based on RCTs and rely at least as much on “consensus meetings” [4]. Fourth, many physicians do not systematically follow guidelines and interventions to support their implementation, which have only small to moderate effects. Fifth, recent years have shown an increased focus on patient-centeredness. It should not be forgotten that the founders of evidence-based medicine already mentioned that evidence-based practice should be the integration of the best available medical evidence, the physician’s expertise, and the patient’s views and preferences [5]. This integration comes close to the terms “research-enhanced health care” or “mindlines” (guidelines-in-the-head, in which evidence from a wide range of sources has been melded with tacit knowledge through experience and continual learning to become internalized as a clinician’s personal guide to practicing in varied contexts) [5, 6]. This also aligns with recent trends to call for more patient-centered approaches in care delivery (e.g., growing interest in patient-reported outcome measures), in care organization (e.g., including experts by experience), and in the selection of research topics (e.g., involving patient organizations in applications for research grants).

The question remains to what degree we base our clinical decisions on this mix of guidelines, physician expertise, and patient’s views and preferences, and to what degree this heterogeneous integration results in patient and caregiver satisfaction. Some examples can illustrate these complexities.

Regarding the question to what degree physicians integrate guidelines, it is interesting to observe that the physician’s choice of an antidepressant seems to be mainly driven by the presence of specific symptoms (52.3%), by wishing to avoid a specific side effect (48.7%), and by the presence of a comorbid condition (45.6%), but much less by the patient’s preference (5.2%) [7]. It remains, however, debatable to what degree the strength of the link between a specific symptom profile and a specific antidepressant is based on solid scientific data, or whether this link is mainly amplified by marketing influences. Another example is that prescription patterns seem to change dramatically over time without changes in guidelines. A Danish study including 197,615 depressed patients showed that between 1996 and 2015, the prescription of selective serotonin reuptake inhibitors (SSRI) decreased while the prescription of serotonin and norepinephrine reuptake inhibitors (SNRI) increased, and that the percentage of psychotherapeutic approaches somewhat increased [8]. These changes in prescription patterns are not the consequence of changes in Danish guidelines but could be the consequence of patent loss (SSRIs were launched earlier than SNRIs and hence lost their patents earlier). In the subgroup of patients with treatment-resistant depression (TRD), the prescription of antipsychotics and especially the use of psychotherapy dramatically increased, although the latter is not often mentioned in published guidelines. The rise in the use of psychotherapy (especially in TRD) could be partially explained by the fact that in the first study years, registration was not always complete [8].

Regarding the role of the expertise of the treating physician, the rate of hospitalization of patients presenting in a psychiatric emergency room varied substantially among different clinicians (between 27.3% for the physician with the lowest to 51.8% for the physician with the highest hospitalization rate): more experienced clinicians and clinicians who feel more comfortable and more connected with the patients had the lowest hospitalization rates [9].

Regarding the patients’ views and preferences, there is, despite the call for patient-reported outcome measures, a discrepancy between what is assessed in RCTs and what patients consider to be important outcome measures. Depressed patients consider the recovery of positive mental health (optimism, vigor, and self-confidence) as the most important treatment goal, but standard depression rating scales do not comprise items covering hedonic tone or positive mood [10]. Moreover, only 55% of patients classified as remitted according to observer-rating scales consider themselves in remission, again underscoring the discrepancy between physicians’ and patients’ views [10]. Misunderstandings between patients and clinicians are more common than imagined by clinicians. In a qualitative primary care study, misunderstandings were found in 82% of consultations, and these seemed to be associated with patients’ lack of participation in the consultation and were often based on inaccurate assumptions by both doctors and patients: doctors seemed unaware of the relevance of patients’ views on successful prescribing and of the fairly widespread aversion to taking medicines [11]. It has also been shown that the divergence in treatment expectations, including which symptoms the physician and the patient rate as priority in treatment goals, significantly predicts outcomes 6 months later, probably illustrating the importance of the doctor–patient relationship [12]. The need to improve the doctor–patient relationship is further substantiated by the finding that 15% of patients admit having secretly recorded a visit to a doctor, and another 11% admit knowing someone who did this; moreover, 35% would consider secretly recording a visit, and another 34% would consider recording a visit after asking permission [13]. Patients reported that the recording was mainly driven by the desire to enhance their understanding of the encounter and, in addition, to share their experience of the encounter with others. The reason for secretly recording was mainly driven by the fear of refusal and was sometimes motivated by prior negative experiences and the wish to have verifiable evidence of such an experience, should it recur; the latter can indeed be understood as suboptimal trust and seems far away from SDM [14].

This narrative review differs from previously published papers since it aims to critically examine SDM in depression as a dynamic, relational, and personalized process rather than a universal ethical standard.

## Methods

This narrative review was conducted to critically examine the concept of SDM in patients with depression. A literature search (limited to papers published in English) was performed in PubMed and PsycINFO using terms such as “depression,” “evidence-based practice,” “personalized medicine,” “shared decision making,” “therapeutic alliance,” and “barriers to SDM.” Both empirical and conceptual publications were included, and the findings were reviewed to critically reflect on key tensions and variability in SDM, including conceptual ambiguities and different interpretations across the literature, the divergence between ethical ideals and clinical realities, as well as variability in patient preferences.

## Results

### SDM: “one size does not fit all”

SDM is often referred to as “no decision about me, without me” and has been endorsed as the gold standard of patient–clinician interaction in preference-based care by the National Academy of Medicine in the Unites States and the National Institute for Health and Care Excellence in the United Kingdom. It can be considered as an intermediate approach between a paternalistic approach (doctor-driven) and a patient autonomy approach (informed approach/consumerism): both clinician and patient share information, both express preferences, and both agree (or agree to disagree) on the decision in an interactive relationship. While this “gold standard” has become an ethical imperative, more recently concerns have emerged. SDM is considered as a health communication model promoting patient-centered care. Inconsistent definitions, concepts, measurement tools, and lack of sufficient evidence for the effectiveness of SDM interventions are contributors to the limited use of SDM [15].

SDM can be considered as an umbrella concept covering therapeutic alliance and physician and patient preferences, information giving, information retrieving, information receiving, patient involvement in goal setting, decision-making, and treatment choice [16, 17] (Table 1). It often goes beyond the psychiatrist–patient dyad, since it sometimes includes multiple care practitioners or multiple sources of information (primary care physician, psychiatrist, psychotherapist, nurse, case manager, the patient and his/her significant others, and including digital tools like ChatGPT or DeepSeek). SDM research has largely focused on the provision of high-quality clinical information from doctors to patients to the neglect of what may be the most important and transformative aspect of SDM: the doctor–patient relationship itself [18]. Another problem concerns the lack of consensus on what should be its finality: improving “outcome” (adherence, symptomatic improvement, functional improvement, and treatment satisfaction), improving patient knowledge, increasing patient participation, or increasing the fit with the patient’s desired level of participation.

**Table 1.** Shared decision-making (SDM): A clinical roadmap (modified from [43]) Table 1. long description.

**General**
How much information do you want on the diagnosis and on the treatment options (from only essential information to exhaustively detailed information)? How much do you want to be involved in the decision-making? Explaining the equality of therapeutic alliance (evidence, experience, preference for both parties)
**Process**
Disclosure of diagnosis and capturing the patient’s personal experience of living with depression Eliciting the patient’s expectations and goals (and their prioritization or personal meaningfulness) Informing about treatment options (exercise, psychotherapy, pharmacotherapy, and neuromodulation) Informing about the benefits and risks of the options Investigation of the patient’s understanding Negotiating between both parties’ preferences Reaching the desired level of share decision-making

Moreover, there is a poor correlation between providers and patient perception of SDM [19]. The subjective perception of participation (by the patient) and objective ratings of SDM behavior (by observers) are often nonaligned. A study investigating the concordance between the observer score for SDM (scored based on videotaped physician–patient consultation) and the patients’ perception of the communication in terms of SDM found virtually no correlation (*R* = −0.01; *P* = .93) and when dichotomizing the observer’s and the patient’s judgment on the observed/experienced SDM (present, absent), 59% of judgments were nonaligned: in 22% of consultations, there was full engagement (SDM present and positive subjective perception); in 38%, there was simulated engagement (SDM present and negative subjective perception); in 21%, there was assumed engagement (SDM absent and positive subjective perception); and in 19%, there was nonengagement (SDM absent and negative subjective perception) [19]. The trusting relationship appeared to be as important as the communication style. Cultural differences are also well known: a report from the Organisation for Economic Co-operation and Development found that the percentage of patients reporting having been involved in decisions about care or treatment was on average 87% for all involved countries but with significant differences between countries (97% in Switzerland, 93% in Australia and Belgium, 91% in France and Luxemburg, 90% for Czechia, the Netherlands, and Canada, 87% in Norway, 85% in Slovenia and Spain, 78% in Greece, 77% in Iceland and Portugal, and 66% in Wales) [20]. Interestingly, the six countries ranked lowest for “patient involvement in decisions about care or treatment” had also the lowest ranking for “trust in healthcare professionals” [20]. It should not be forgotten that not all patients prefer SDM: a systematic review of 115 studies showed that 63% of patients prefer participating in decisions, 21% prefer delegating decisions, and 16% had mixed opinions [21].

### SDM in mental health care: A personalized approach

The literature suggests that SDM is lower in mental health care compared to physical health care [22, 23]. It was reported that in community mental health care, only 28% of patients reported “yes, definitely” when they were asked whether they were involved in the medical decision-making (while this was 80.4% in oncology, 75.2% in maternity care, and 61.6% in primary care settings) [24, 25]. Another 42% reported that they were involved to some extent, and 28% reported they were not involved at all. No changes were observed in the percentage of patients feeling involved in treatment decisions between 2014 and 2022. A systematic review on the use of SDM in 150 collaborative care programs for patients diagnosed with depression revealed that only in 29% of the programs, SDM was explicitly mentioned, and, when there was implementation, it was often by the primary care physician (60%) and much less by the psychiatrist (29%). In 90% of the programs, the focus of SDM was on a single decision (choice or treatment) [26]. No significant differences were found in programs developed in 2000–2009 or in 2010–2019. This is in contrast with the evolution of patient expectations: a systematic review found that the preference for shared or autonomous decision-making was 43% in studies before 1990, 51% in studies from 1990 to 1999, and 71% in studies published after 1999 [21].

Two major problems with SDM in mental health care are that it is often treated and measured as a universal attitude (despite patients differing in terms of their desired level/degree of SDM) and that it is viewed as a static snapshot rather than as a dynamic, ongoing process (Table 2).

**Table 2.** SDM: personalization and process (during acute, continuation, and maintenance treatment phase) Table 2. long description.

Personalization	Process
*– Sociodemographic and cultural characteristics*	*– Sociodemographic and cultural characteristics*
– Patient’s desire for knowledge	– Actual treatment experience (efficacy, side effects)
– Patient’s desire for degree of SDM	– Growth in therapeutic alliance trust
– Goal setting	– Changes in goal setting
– Clinical status (severity, suicidality, suspiciousness, and lack of insight)	– Changes in clinical status (changing trust in one’s own competence, in desire for autonomy)

Expectations vary among individuals, and a balancing act of being attuned to patients’ needs is recommended: patients differ in knowing (knowing or not knowing: the need for information) and in wanting to know (wanting or not wanting to know: the need for autonomy), as they differ in the level of SDM they desire. It is not so much a “one size fits all” approach (medication leaflets reflecting a biomedical approach), but rather a “tailoring” approach (differentiated information giving, taking patient preferences for information into account). A scoping review on the information needs of patients, including 52 studies, showed that the patients’ reported preferred information sources were friends and relatives (100%), general practitioners (80%), broadcast media (78%), educational programs (75%), the internet (67%), and only 20% preferred mental health professionals. In practice, the current information sources are the internet (84%), general practitioners (84%), friends and relatives (75%), and mental health professionals (39%) [27]. The most frequently reported information needs included strategies to cope with depression and alleviate symptoms/self-management (76.5%), other people’s experience of depression in general (66.7%), treatment options and comparison between options (63.2%), available local services (58.3%), and side effects of treatment (50.0%) [27]. In patients taking recently prescribed antidepressants, only 59% were overall satisfied with the information about the medication: satisfaction was 81% for “information delivered with kindness and respect” but only 59 and 56% for “sufficiently informed to co-decide about antidepressant treatment” and “sufficiently informed to co-decide about side effects,” respectively [27]. As a group, patients prefer to know about severe side effects both with high and low frequency, while they often prefer to know about mild side effects only when they occur frequently [27]. A more personalized approach, asking the patient whether he or she desires to know about possible side effects (severity and frequency) before explaining them in more detail, may well decrease patients’ desire for side effect information, and hence eventually avoid nocebo effects [28]. Providing patients with comprehensive information about their medication is essential in facilitating patient autonomy. However, informing about side effects might also cause harm (nocebo effect). To handle this ethical dilemma, promising approaches targeted to reduce expectation-induced side effects (nocebo) in a manner that is personalized to the patient’s characteristics, underlying disease, health status, and informational needs while still respecting patient autonomy and truthfulness: validated strategies include framing side effect information positively; personalizing the information to the patient’s characteristics, underlying disease, health status, and informational needs; educating about the medication’s mechanism of action; and explicitly informing about the nocebo effect itself [28]. Although most patients want to discuss treatment options, they may not wish to make the final decision. Most or almost all patients want their physician to know them as a person, and they want disclosure of treatment choices (effectiveness, side effects), but only 39% want to personally make the final selection of the treatment [29]. Female gender, higher education, better self-rated health, fewer medications, and a shorter relationship with the treating physician predicted higher scores on self-selection of treatment choice. Several patient decision aids have been developed, but they mainly focus on the different side effect profiles of antidepressants. An example is the patient decisional aid (PDA), developed for TRD, where the advantages (how well do treatments work) and disadvantages (how often do certain side effects occur, factors that may impact convenience, and financial considerations) of three treatment modalities (electroconvulsive therapy (ECT), esketamine nasal spray, and transcranial magnetic stimulation) are compared so that the patient can make a more informed preference [30]. Patients exposed to this decisional aid showed, compared with patients not exposed, a significant decrease on the Decisional Conflict Scale (from 42.2 to 28.1) and a significant increase on the self-efficacy scale (from 86.0 to 95.5); it should be noted that these were patients suffering from TRD but able to give informed consent [30]. A problem with “informed consent” is, of course, that the provided information can be biased by the clinician’s opinions or by the clinical researcher’s interest in enrolling patients into ongoing studies. Another example is the Psymatic Treatment Optimizer, which is a tool helping to choose an antidepressant taking into account the specific side effect profile (the tool provides a heatmap where dilemmas between one or other side effect can be solved in a collaborative way) [31].

Expectations also vary over time depending on the clinical status of the patient, the actual experience with the treatment, and the growth in the therapeutic alliance.

First, the psychiatrist’s approach can and should vary depending on the clinical status of the patient. In cases of suicidality or hampered decision capacity (due to severe symptoms, important suspiciousness or passivity, limited intellectual capacity, lack of insight, low health literacy, and psychotic or schizoaffective depression), a more paternalistic decision-making or even a substituted decision-making, which can be explicit and formal (with involuntary commitment as extreme positioning) or more often implicit and informal, may be necessary. However, when the clinical status asks for a more active role of the health care provider, one should differentiate between trying to persuade a patient (understood as aiming for the patient to consent to a certain treatment, although he/she prefers not to), trying to convince him/her (understood as aiming for the patient to ‘want’ the treatment), and recommending (the doctor merely providing his/her professional opinion). The first attitude is commonly considered as paternalistic, while the latter two are generally not paternalistic and can become closer to a SDM attitude [32]. Expectations from treatment also vary depending on the antidepressant treatment phase: in the continuation treatment phase, patients attach more attention to the return of positive mood than in the acute treatment phase [12].

Second, having actual experience with a particular treatment can influence expectations. A study investigated the relative importance of different aspects of treatment (treatment efficacy, time to full response, transient post-dose issues (like dissociation), risk of ulcerative cystitis, risk of cognitive impairment) in a clinical trial with patients treated with esketamine and patients from an online panel suffering from TRD [33]. Thirty-five percent of clinical trial patients, but only 14% of panel patients, chose the treatment with greater improvement regardless of side effects or risks. Moreover, 11% of the former and 22% of the latter were concerned with transient post-dose issues (e.g., dissociation). Interestingly, only 1.3% of the former and 5.4% of the latter selected “time to full response” as the dominant driver in choosing the treatment [33].

Third, the preferred doctor–patient relationship and the preferred role (active, collaborative, or passive) can change over time. In patients initiated for a left ventricular assist device, roughly half of the patients changed their stated preference (active, collaborative, or passive) from one category to another over 6 months, with a slight increase in the percentage preferring a more active role [34]. It has been observed that the preferred role changes with the stage of the patient–provider relationship. A study in primary care, including 1168 patients diagnosed with depression, showed that the degree of perceived SDM was 50.7 (on a score between 0 and 100): perceived SDM decreased when treatment duration was longer than 6 weeks and was lower in older patients, again underscoring the dynamic process of SDM [35]. Changes in trust in the healthcare provider, changes in trust of one’s own competence, in desire for autonomy, and in perceived self-efficacy occur frequently during a longer doctor–patient relationship [36]. A kind of “guarded alliance” has also been described: trust and cooperation do exist but are limited, cautious, or fragile, and not only depend on the actual health care provider–patient interaction (alliance and communication) but also on past experiences in medical care, trauma, personality issues, and attachment style [36]. A history of trauma (even when denied during an initial assessment) is sometimes shared only after multiple encounters (growing alliance) and can be another dynamic in the SDM asking for more specific treatments.

### Effects of SDM on treatment outcome

The question remains what the finality of SDM should be: improving “outcome” (adherence, symptomatic improvement, functional improvement, and treatment satisfaction), or improving knowledge of the patient, increasing patient participation, or increasing the fit with the desired level of participation. Moreover, the many reported SDM interventions have different ingredients, hampering final conclusions.

A systematic review focusing on studies evaluating SDM (using decision aids) in mood disorders found that all of them effectively improved patient satisfaction and engagement in the decision-making process, and all showed significant improvement in depression outcomes and medication adherence [37].

However, a Cochrane review of “randomized clinical trials” of SDM versus SDM concluded that there is generally a low level of certainty about whether SDM interventions improve clinical outcomes, users’ overall satisfaction, users’ overall knowledge, and treatment continuation [38]. It should, however, be noted that the included studies were difficult to compare: some had digital interventions, others had a decision aid or peer-co-led educational programs, or guided self-determination or multifaceted blended eHealth interventions, or medication review tools hampering global conclusions. Moreover, a major problem with most studies investigating the effect of SDM on outcomes is the difficulties regarding randomization: even in the control arm, some doctor–patient interactions will be more or less SDM.

A more recent meta-analysis of 23 depression studies investigating outcome and drop-out in depressed patients depending on methodologically strict preference trials (limited to (1) doubly randomized preference trials, where first patients are randomized to choice versus no choice, and then one arm can choose between two treatments or (2) fully randomized preference trials, where preference is assessed at baseline before all patients are randomized to a match or a mismatch treatment arm) found a small Cohen’s delta of 0.17 for depression outcome but a large delta of 1.65 for completing the treatment [39]: The effect on adherence was stronger than the effect on outcome (43% of the included trials were focusing on the choice between antidepressants and psychotherapy, and another 37% on the choice between different forms of psychotherapy).

Another recent literature review investigating the effect of the therapeutic alliance (TA – defined as shared goals, agreement on interventions, therapeutic bond with the development of personal connection, trust, and a sense of safety) through quantitative assessment in patients with major depressive disorder found that TA predicted clinical outcome in MDD patients: higher alliance scores predicted better outcomes, and greater improvement predicted a higher alliance [40]. Interestingly, the patient’s perception of TA tended to be a stronger predictor of clinical outcomes compared to assessments made by therapists or external observers.

Some studies focused on more specific outcome variables. The use of a PDA (focusing on pros and cons of options; understanding how treatments were administered, possible side-effects, and likelihood of benefit) for TRD has been shown to result in a significant decrease on the Decisional Conflict Scale and a significant increase on the Decisional Self-Efficacy Scale [30]. Another within-group study found that a high SDM score resulted in a four times higher implementation of treatment recommendations: the items showing the strongest correlations were “giving the opportunity to ask questions or voice concerns” (OR: 4.95), “being asked about any problem or side effects from my treatments,” (OR: 2.75), and “being involved in deciding on my treatment plan” (OR: 2.40) [41].

### Barriers toward implementation of SDM

Implementation of SDM in healthcare systems has proved difficult and slow, including in psychiatric practice, because of barriers in healthcare providers and in patients [42]. A clinical roadmap for implementing SDM in patients with depression is given in Table 1.

Commonly reported barriers in physicians include misconceptions about the nature of SDM, the lack of published evidence of efficacy in mental health conditions, time pressure, the belief that SDM is inappropriate for patients with (severe) mental disorders, beliefs about poor health literacy, and patients’ insight. In addition, clinicians “imagine” they already involve patients in decision-making but the patients do not agree that they have been appropriately involved [42]. A qualitative study found that the greatest barrier was the overarching theme of disease-centeredness comprising four essential beliefs negatively interfering with SDM: the doctor is the expert, the disease determines the treatment, there is one best treatment for each disease, and the doctor is responsible for decisions [43].

While most patients “tell” they prefer a more collaborative role when facing psychiatric medications and a more autonomous role for decisions related to psychosocial interventions in medical encounters, they are often passive, revealing ambivalence in their attitude [44]. The fact that people with chronic illnesses are less likely to prefer an active role can at least partially be explained by the fact that they have learned to assume a more passive role. Moreover, having choices can increase the sense of lost opportunities; patients may have difficulty valuing outcomes, and choice may lead to dissatisfaction when expectations are not met [44].

## Conclusion

SDM is rather ill-defined but can be considered as a kind of umbrella concept covering therapeutic alliance and physician and patient preferences, information giving, information retrieving, information receiving, patient involvement in goal setting, decision-making, and treatment choice. SDM in depression should move away from an ethical imperative where one size fits all toward a more personalized approach, tailoring the medical encounter to the physician’s and patient’s expectations. It cannot be conceived as a “snapshot” since it is an ongoing dynamic process where adjustments should be made depending on the growth of the doctor–patient relationship and the clinical status. The clinician should be aware of barriers to effective implementation of SDM in him or herself, in the patient, and in the significant others. The different definitions of SDM and the many reported SDM interventions (having different ingredients) hamper final conclusions on the effect of SDM on outcomes.

## Long descriptions

### Table 1 Long description

The table is structured into two main vertical sections.

General section includes:

* Inquiry into the patient’s desired level of information regarding diagnosis and treatment options, ranging from essential to exhaustive.

* Inquiry into the patient’s desired level of involvement in decision-making.

* Explanation of the equality of the therapeutic alliance, considering evidence, experience, and preferences for both parties.

Process section includes:

* Disclosure of diagnosis and capturing the patient’s personal experience with depression.

* Eliciting and prioritizing the patient’s expectations and goals.

* Informing the patient about treatment options including exercise, psychotherapy, pharmacotherapy, and neuromodulation.

* Informing the patient about the benefits and risks of these options.

* Investigation of the patient’s understanding.

* Negotiating between the preferences of both parties.

* Reaching the desired level of shared decision-making.

Navigate back to Table 1.

### Table 2 Long description

The table is divided into two main columns.

Column 1 is titled Personalization. It includes the following bulleted items.

* Sociodemographic and cultural characteristics.

* Patient’s desire for knowledge.

* Patient’s desire for degree of S D M.

* Goal setting.

* Clinical status including severity, suicidality, suspiciousness, and lack of insight.

Column 2 is titled Process. It includes the following bulleted items.

* Sociodemographic and cultural characteristics.

* Actual treatment experience including efficacy and side effects.

* Growth in therapeutic alliance trust.

* Changes in goal setting.

* Changes in clinical status including changing trust in one’s own competence and in desire for autonomy.

Navigate back to Table 2.
